# Genetic Diversity of 17 Autochthonous Italian Chicken Breeds and Their Extinction Risk Status

**DOI:** 10.3389/fgene.2021.715656

**Published:** 2021-09-14

**Authors:** Dominga Soglia, Stefano Sartore, Emiliano Lasagna, Cesare Castellini, Filippo Cendron, Francesco Perini, Martino Cassandro, Margherita Marzoni, Nicolaia Iaffaldano, Arianna Buccioni, Sihem Dabbou, Annelisse Castillo, Sandra Maione, Chiara Bianchi, Margherita Profiti, Paola Sacchi, Silvia Cerolini, Achille Schiavone

**Affiliations:** ^1^Dipartimento di Scienze Veterinarie, Università degli Studi di Torino, Turin, Italy; ^2^Dipartimento di Scienze Agrarie, Alimentari e Ambientali, Università degli Studi di Perugia, Perugia, Italy; ^3^Department of Agronomy, Food, Natural Resources, Animals and Environment (DAFNAE), Università di Padova, Viale dell’Università, Legnaro, Italy; ^4^Dipartimento di Scienze Veterinarie, Università di Pisa, Pisa, Italy; ^5^Dipartimento Agricoltura, Ambiente e Alimenti, Università degli Studi del Molise, Campobasso, Italy; ^6^Dipartimento di Scienze e Tecnologie Agrarie, Alimentari, Ambientali e Forestali, Università di Firenze, Florence, Italy; ^7^Center Agriculture Food Environment (C3A), University of Trento, Trento, Italy; ^8^Research and Innovation Center, Fondazione Edmund Mach, San Michele all’Adige, Italy; ^9^Dipartimento di Medicina Veterinaria, Università degli Studi di Milano, Lodi, Italy

**Keywords:** genetic resources, local poultry breeds, microsatellites, genetic variability, conservation

## Abstract

The preservation of genetic variability of autochthonous poultry breeds is crucial in global biodiversity. A recent report revealed small breed size and potential risk of extinction of all native Italian poultry breeds; therefore, a correct assessment of their genetic diversity is necessary for a suitable management of their preservation. In this work, we provided an overview of the contribution to poultry biodiversity of some Italian autochthonous breeds reared in conservation centers devoted to local biodiversity preservation. The level of genetic diversity, molecular kinship, inbreeding, contribution to overall genetic diversity, and rate of extinction of each breed were analyzed with a set of 14 microsatellite loci in 17 autochthonous chicken breeds. To evaluate genetic variability, total number (Na), and effective number (Ne) of alleles, observed (Ho) and expected (He) heterozygosity, and *F* (Wright’s inbreeding coefficient) index were surveyed. The contribution of each analyzed breed to genetic diversity of the whole dataset was assessed using MolKin3.0; global genetic diversity and allelic richness contributions were evaluated. All the investigated loci were polymorphic; 209 alleles were identified (94 of which private alleles). The average number of alleles per locus was 3.62, and the effective number of alleles was 2.27. The Ne resulted lower in all breeds due to the presence of low-frequency alleles that can be easily lost by genetic drift, thus reducing the genetic variability of the breeds, and increasing their risk of extinction. The global molecular kinship was 27%, the average breed molecular kinship was 53%, and the mean inbreeding rate 43%, with a self-coancestry of 78%. Wright’s statistical analysis showed a 41% excess of homozygous due to breed genetic differences (34%) and to inbreeding within the breed (9%). Genetic variability analysis showed that 11 breeds were in endangered status. The contribution to Italian poultry genetic diversity, estimated as global genetic diversity, and ranged from 30.2 to 98.5%. In conclusion, the investigated breeds maintain a unique genetic pattern and play an important role in global Italian poultry biodiversity, providing a remarkable contribution to genetic variability.

## Introduction

In the last decade, many developed countries recorded a raising interest toward local breeds and traditional products (Franzoni et al., 2021); the most likely explanation of this new tendency may be due to the fact that the farming system of local breeds, extensive and therefore more sustainable, is perceived to be more respectful of animal welfare and environment compared to intensive industrial farming (Soglia et al., 2017, 2020). In the past, local breeds were massively substituted by commercial hybrids able to provide 67% of meat and 55% of egg production given their higher aptitude for growing and laying. Although the introduction of these strains lowered the size of native chicken breeds, thus threatening their existence (Hillel et al., 2003; Granevitze et al., 2007; Chen et al., 2008), they are important reservoirs of biodiversity for future needs in a scenario of environmental change, and moreover, they still have some potential economic profit [Food and Agriculture Organization (FAO), 2015]. Nevertheless, most of the genetic investigations were focused on commercial lines so far [Food and Agriculture Organization (FAO), 2011]; in fact, because of their low commercial performances, local chicken breeds are hardly matter of interest, and far less attention was given to genetic conservation of these resources compared to other livestock species such as cattle and sheep so far (Blackburn, 2006; Wilkinson et al., 2011). In this regard, a main interest is represented by the assessment of the genetic variability of native breeds to start adequate and reliable conservation programs. Biodiversity preservation is a crucial target for the survival of local breeds that can be achieved with a specific strategy based on mating schemes and consistent checking of genetic variability data. To define priorities is essential to assess the conservation value of each breed and to manage the genetic diversity. It is paramount to take into account that conservation priorities may significantly differ according to the weight given to within- and between-breeds genetic diversity (Ginja et al., 2013).

Mathematical software, such as GenAlEx v6.501 (Peakall and Smouse, 2012) and MolKin v3.0 (Gutiérrez et al., 2005), and the methods proposed by Caballero and Toro (2002)are useful tools to estimate diversity parameters that can then help in taking decisions on conservation and management of these native chicken breeds. In particular, MolKin v3.0 allows estimating the contribution to genetic diversity given by both within- and between-breeds variability.

In this work, we aimed to obtain an overview of the contribution of 17 Italian chicken breeds included in the Registry of Indigenous Poultry Breeds and in the project TuBAvI ‘‘Conservation of Biodiversity in Italian Poultry Breeds’’ (^1^ Castillo et al., 2021) to Italian poultry biodiversity and their conservation risk status. This investigation was performed with a set of 14 microsatellite loci chosen from the group of 30 markers suggested by the international society of animal genetics (ISAG-FAO) to assess the poultry genetic diversity [Food and Agriculture Organization (FAO), 2011; Soglia et al., 2017]. Microsatellites are a reliable tool to investigate the genetic variability due to their high polymorphism, the codominant inheritance, and the even distribution throughout the genome (Cheng et al., 1995) and were used in other autochthonous chicken breeds (De Marchi et al., 2005a,b; Tadano et al., 2007; Zanetti et al., 2010, 2011), although some results were also obtained from the investigation of the mt-DNA polymorphism in some European chicken breeds (Revay et al., 2010; Englund et al., 2014). Moreover, in Malomane et al. (2019) with the aid of a single-nucleotide polymorphism (SNP) investigation, provided an interesting picture of the genetic variability of 201 chicken breeds spread all over the world and included the Padovana breed; more recent articles (Cendron et al., 2020, 2021) reported a markedly different level of genomic inbreeding among Italian chicken breeds based on runs of homozygosity (FROH). We investigated the level of genetic diversity, molecular kinship, inbreeding, contribution to overall genetic diversity, and rate of extinction of each breed. In order to evaluate the breed genetic variability, total (Na) and effective number (Ne) of alleles, observed (Ho) and expected (He) heterozygosis, and *F* (Wright’s inbreeding coefficient) were surveyed. The collected information could be useful for planning new safeguarding strategies.

## Materials and Methods

### Samples

Blood samples were collected in compliance with the European rules [Council Regulation (EC) No. 1/2005 and Council Regulation (EC) No. 1099/2009]. Blood samples were collected during routine health controls by the public veterinary service.

A total of 645 samples were collected from 17 different autochthonous chicken breeds (Ancona, Bianca di Saluzzo, Bionda Piemontese, Livorno Bianca, Livorno Nera, Mericanel della Brianza, Mugellese, Ermellinata di Rovigo, Millefiori di Lonigo, Padovana, Polverara Nera, Pepoi, Robusta Lionata, Robusta Maculata, Romagnola, Siciliana, and Valdarnese), reared in conservation centers devoted to local genetic preservation (Figure 5). All breeds are officially recognized by the Italian authorities and by the FAO.

**FIGURE 1:**
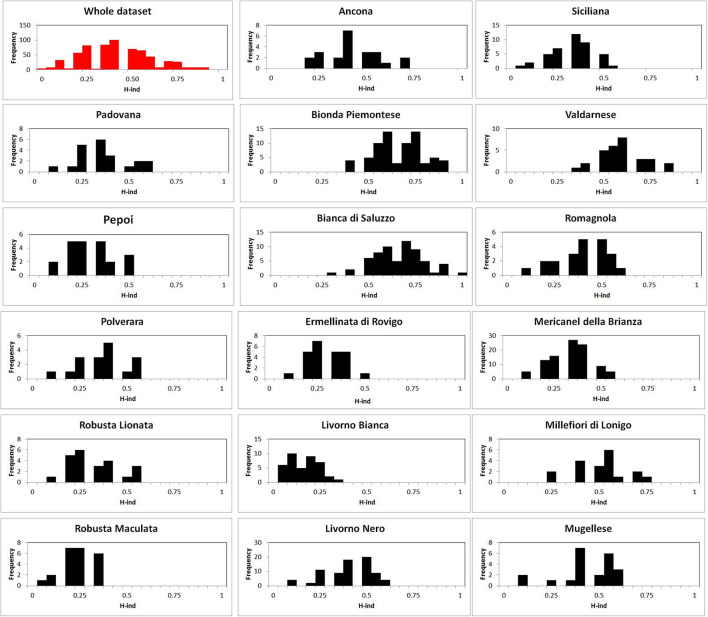
Graphical output of individual heterozygosity value (Hind) distribution: in red whole dataset analysis, in black breed analysis.

**FIGURE 2:**
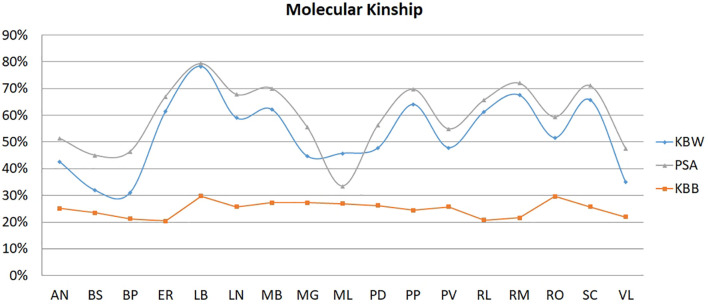
Graphical output of molecular kinship. AN, Ancona; BP, Bionda Piemontese; BS, Bianca di Saluzzo; ER, Ermellinata di Rovigo; LB, Livorno Bianca; LN, Livorno Nera; MB, Mericanel della Brianza; MG, Mugellese; ML, Millefiori di Lonigo; PD, Padovana; PP, Pepoi; PV, Polverara; RL, Robusta Lionata; RM, Robusta Maculata; SI, Siciliana; VA, Valdarnese; RO, Romagnola; *K*_BW_, within-breed kinship; PSA, proportion shared allele, and *K*_BB_, between-breed kinship.

**FIGURE 3:**
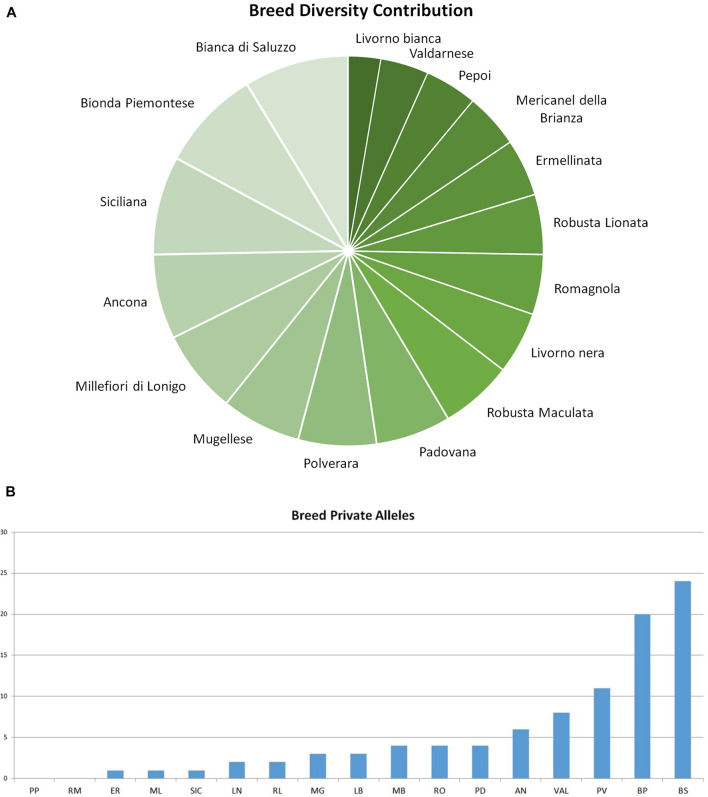
Graphical output of breed diversity contribution. **(A)** Breed contribution to overall genetic diversity. **(B)** Breed private alleles: bar graph of private allele number detected in each breed. AN, Ancona; BP, Bionda Piemontese; BS, Bianca di Saluzzo; ER, Ermellinata di Rovigo; LB, Livorno Bianca; LN, Livorno Nera; MB, Mericanel della Brianza; ML, Millefiori di Lonigo; MG, Mugellese; PD, Padovana; PP, Pepoi; PV, Polverara; RL, Robusta Lionata; RM, Robusta Maculata; SI, Siciliana; VA, Valdarnese; and RO, Romagnola.

**FIGURE 4:**
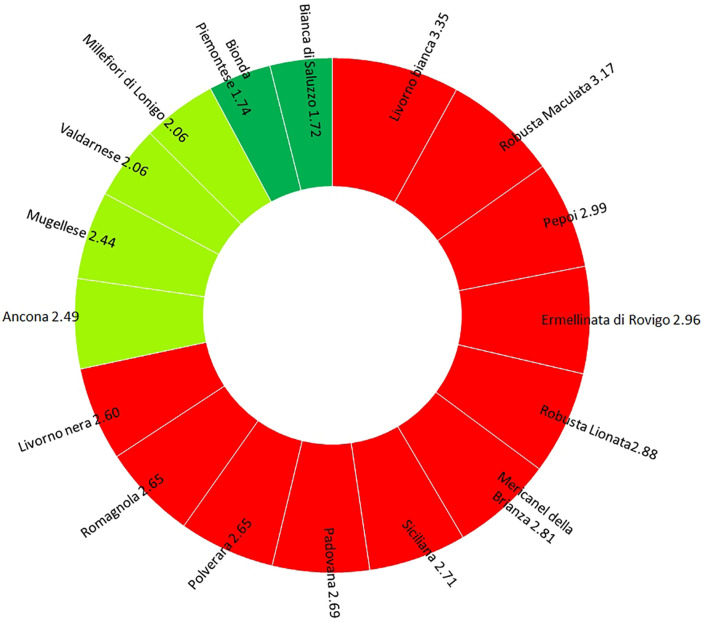
Graphical output of Extinction Risk Index (ERI): breeds with ERI > 2.5 (red), breeds with ERI = 2–2.5 (light green), and breeds with ERI < 2 (green).

**FIGURE 5:**
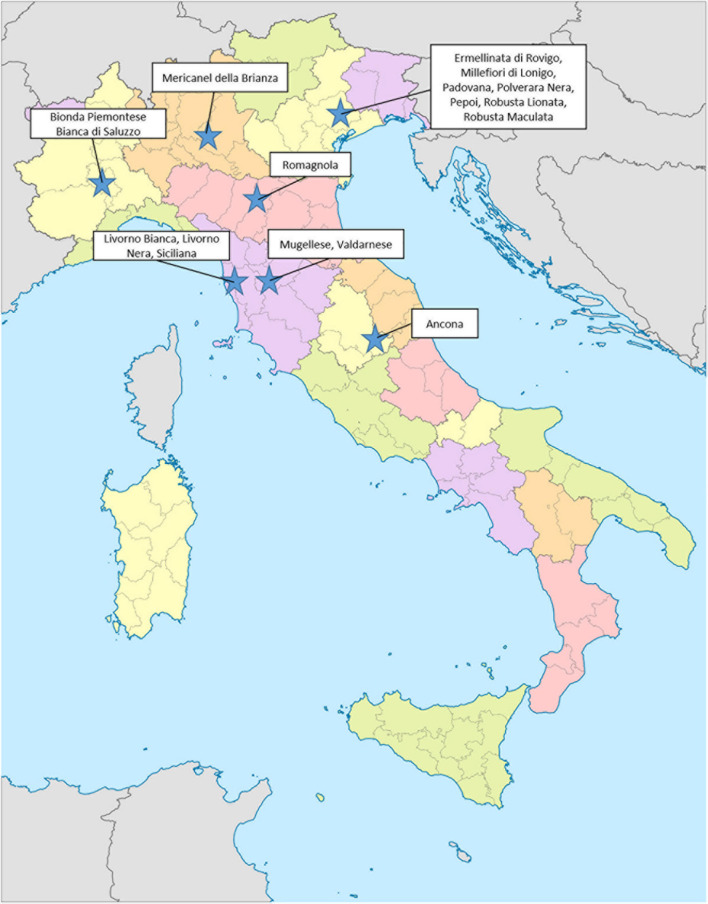
Geographic localization of genetic conservation centers.

All blood samples (about 2 mL) were collected from ulnar veins and stored in Vacutainer^®^ tubes containing EDTA as an anticoagulant; a blood aliquot was immediately frozen at −20°C pending DNA analysis. DNA was extracted with the NucleoSpin^®^ Blood QuickPure kit (Macherey-Nagel, Düren, Germany).

The experimental protocol was approved by the Bioethical Committee of the University of Turin (protocol no. 451944).

### DNA Genotyping

The genetic characterization was done using a panel of 14 microsatellites (Supplementary Table 1) chosen for their high polymorphic content (number of alleles and heterozygosity); this set of 14 markers has already shown its reliability in a previous survey carried out by Soglia et al. (2017). Multiplex polymerase chain reaction (PCR) and amplicons processing were carried out according to Sartore et al. (2014). The markers were subjected to a multiplex PCR amplification in 10-μL reactions using the following final concentrations: 1X buffer Qiagen (Hilden, Germany), 0.4 mM dNTPs, and 0.05 mM HotStart Taq Qiagen. The following thermocycling conditions were used: an initial denaturation step of 15 min at 95°C, 31 cycles of 30 s at 95°C, 1 min at the annealing temperature specific to of each multiplex PCR, 1 min at 72°C, and a final extension of 7 min at 72°C. Analyses of fragments were performed using the automated DNA Genetic Analyzer ABI PRISM 310 (Applied Biosystems, Foster City, CA, United States) and the computer software GeneMapper 4.0 (Applied Biosystems). Allele calling was adjusted to Aviandiv project^2^ nomenclature including nine DNA reference samples. An error assay was performed by replicating the genotyping on a randomly chosen 10% of individual samples (Pompanon et al., 2005).

### Genetic Diversity and Allelic Richness

Genetic variability analysis was computed using GenAlEx v6.501 (Peakall and Smouse, 2012) and MolKin v3.0 (Gutiérrez et al., 2005). The polymorphic information content (Polymorphic Information Content (PIC), Botstein et al., 1980) at both marker and breed level was computed (Guo and Elston, 1999). The PIC refers to the value of a marker for detecting polymorphism within a breed, depending on the number of detectable alleles, and the distribution of their frequency. In MolKin, most variables were computed weighting information provided for each locus by its PIC. All genetic parameters were estimated per locus and across all loci for each breed, on global dataset, and as breed average value. The average number of alleles per locus (Na) and the allelic richness (Rt), and using the rarefaction method, were reported: Rt is the normalized allele size of the breed; the normalized population size was computed on the lower breed size in the dataset considering only those individuals genotyped for all markers (Hurlbert’s, 1971 rarefaction method); the effective allele number (Ne) was estimated as the number of equally frequent alleles in an ideal breed, and to simplify significant comparisons of allelic diversity across loci with dissimilar allele frequency distributions. The number of private alleles (Np) was counted. Also, Ho and He were estimated and *F* [Wright’s inbreeding coefficient: 1 − (Ho/He)] was calculated as deviation from expected heterozygosity. The global tests across populations and loci using Guo and Thompson 1992 were performed in GenePOP (Raymond and Rousset, 1995; Rousset, 2008) and *P*-values obtained using a Markov chain of 10,000 dememorization steps, 500 batches, and 5,000 interactions. The frequency of null alleles was estimated by FreeNA (Chapuis and Estoup, 2007).

### Molecular Inbreeding

The molecular inbreeding was evaluated using individual observed heterozygous (Hind) as nH/nL where nH is the number of heterozygous loci, and nL is the total number of tested loci. Graphical output of Hind value distribution, global, and for each breed, was performed in GeneAlex to evaluate the quantity and distribution of inbreeding. Indeed, the inbreeding of each breed was evaluated as Ho and statistical analysis was performed: mean, median, standard deviation, standard error (SE), and maximum and minimum values for breed.

In addition, the coefficient of inbreeding of an individual (*F*i) was estimated by the formula *F*i = 2*s*_*i*_ − 1 where *S*_i_ (self-coancestry) is the molecular coancestry of an individual *i* with itself; it is related to the individual homozygosity. Mean self-coancestry for whole dataset (I, inbreeding) and that in each breed (*I*_B_ = breed inbreeding) were estimated.

### Molecular Kinship

The kinship was estimated as molecular coancestry coefficient between individuals included itself (Caballero and Toro, 2002): for each individual, molecular kinship was estimated as average of the molecular coancestry coefficients between the individual and all the other individuals in the whole dataset, whole individual kinship (*K*_IW_), and in breed dataset, breed individual kinship (*K*_IB_). Global kinship (*K*_G_) is the average of all *K*_IW_, whereas within-breed kinship (*K*_BW_) and between-breed kinship (*K*_BB_) are simply computed averaging the corresponding values for all the within-breed (*K*_IB_) or between-breeds pairs of individuals. Additionally, 100 bootstrapping adjusted for 50 sampling size and weighted for PIC was applied. The molecular kinship within breed was also estimated as proportion shared allele (PSA).

### Global Diversity and Breed Contribution

The genetic diversity has been defined in terms of molecular coancestry distances (Eding and Meuwissen, 2001). Global diversity (GD) and breed diversity are computed averaging the corresponding values for all the within- or between-breed pairs of individuals.

### Wright’s *F* Statistics

Wright’s (1978)*F* statistics were used to evaluate the genetic diversity: *F*_IS_ (heterozygote deficiency within breed), *F*_ST_ (heterozygote deficiency due to breed subdivision), and *F*_IT_ (heterozygote deficiency in the total breed) were obtained as in MolKin v.3. The bootstrapping method adjusting for sampling to avoid bias in estimates because of unequal sample sizes and weighted for PIC information was applied (Caballero and Toro, 2002).

### Breed Genetic Diversity Contribution

The contribution of each analyzed breed to global genetic diversity and breed extinction impact was assessed using MolKin3.0 following Caballero and Toro (2002), removing from dataset all breeds except the one whose impact from disappearing was taken into account. In the results, GD was the genetic diversity of the dataset after removing the breed; GD_W_ (within-breed) and GD_B_ (between-breeds) were percentage of lost diversity. The contribution of each breed was evaluated by the formula: GD_T_ = GD_W_ + GD_B_; GD_T_ is the total contribution to GD expressed as GD loss percentage respect to GB. The contribution to GD of each analyzed breed was quantified as 1 − GD_T_; a pie chart was produced. The allelic richness contribution was estimated as Np and plotted for each breed. A positive GD_T_ value of a breed means that in the remaining dataset the GD increases; consequently, the breed with a positive GD_T_ would not be the first choice for a conservation program.

### Conservation Risk Status

For the assessment of the risk of extinction of studied breeds, more genetic variability indices have been taken into account to quantify the inbreeding depression and genetic drift: He and Ho, as estimation of genetic variability and inbreeding, respectively, together with the number of alleles (Na), allelic richness (Ne), and the number of alleles with frequency of less than 5% as an estimate of the risk of total variability reduction. Graphical output of allelic pattern was performed in GeneAlex.

### Extinction Risk Index

Four indices of variability were selected and combined to define an extinction risk index (ERI).


ERI=F+I+BK+BWPSA+O

where, *F* index estimates the excess of homozygous, *I*_B_ means inbreeding, *K*_BW_, PSA gives the proportion of diversity between individuals, and *O* = 1 - Ho, homozygosity. ERIs range between 0 and 5. The mean point 2.5 has been arbitrarily chosen as the reference point to define when a breed should be considered at risk of extinction.

## Results

### DNA Genotyping

A total of 645 animals were analyzed. A total of 209 alleles were identified, and a good level of polymorphism was detected in all investigated microsatellite loci. The set of microsatellites used in the investigation had a mean PIC of 0.66. The mean Na per locus is 15 (5–39), but the mean Ne is 4.35 (1.85–12.78). In eight loci, it is possible to observe a significant excess of homozygosity. The mean *F*_IT_ is 0.40 ± 0.2, and *F*_ST_ is 0.34 ± 0.2. The frequency of null allele estimated by FreeNA was reported in Supplementary Table 3. For microsatellite LEI228, the frequency of null allele was estimated as > 0.10 in 10 breeds. In Ancona and Bianca di Saluzzo, the frequency of null allele was > 0.10 in six loci, whereas in Padovana, the frequency of null allele was > 0.10 in five loci. Global and pairwise *F*_ST_ values (Weir and Cockerham, 1984) estimated with and without correction for null alleles in FreeNA gave similar values (±0.01 SE), suggesting that null alleles do not have a large impact on population differentiation.

### Genetic Diversity

Overall, breed level indices of genetic diversity (Na, Rt, Ne, Ho, and He) varied across breeds (Table 1) according to the following: Na values ranged between 6.71 ± 0.62 and 1.86 ± 0.14 (Rt 5.46–1.80), whereas Ne values ranged between 3.57 ± 0.41 and 1.37 ± 0.10. The mean Na was 3.63 ± 0.13 (Rt = 3.31 ± 0.26), and Ne was 2.27 ± 0.09. The level of genetic diversity based on Na is higher in Bionda Piemontese and Bianca di Saluzzo (6.50 and 6.71, respectively) followed by Valdarnese (5.29) and Ancona (4.36); the lowest value is found in Livorno Bianca (1.86). The mean Ho was 0.43 ± 0.02, and the *F*_is_ index underlines a slight but significant excess of homozygous in most breeds even if the mean value is 0.09 ± 0.02 and ranged between 0.21, in Polverara and −0.04 in Livorno Nera; only in 2 breeds the *F*_is_ resulted to have a significant negative value: Bianca di Saluzzo (−0.03 ± 0.02) and Bionda Piemontese (−0.01 ± 0.02). Bianca di Saluzzo had the highest Na value, whereas Livorno Bianca had the lowest: Na 1.86 ± 0.14, Ne 1.37 ± 0.10, and Ho 0.17 ± 0.04. Among the 14 loci, the number of loci that deviated from HW equilibrium at the breed level ranged from 2 (Livorno Bianca and Romagnola) to 10 (Bionda Piemontese) with an average of 4.88 (*p* < 0.05).

**TABLE 1:** Breed genetic variability parameters.

Breed (N)	PIC	P	Na	Rt	Ne	Ho	He	*F*_IS_	*P*	*I*_B_
Ancona (23)	0.40	100	4.36 ± 0.49	4.22	2.60 ± 0.31	0.45 ± 0.04	0.56 ± 0.04	0.20 ± 0.03	0.0000	0.77
Bionda Piemontese (72)	0.52	100	6.50 ± 0.61	4.89	3.40 ± 0.23	0.68 ± 0.05	0.69 ± 0.02	−0.01 ± 0.02	0.0009	0.65
Bianca di Saluzzo (64)	0.54	100	6.71 ± 0.62	5.46	3.57 ± 0.41	0.69 ± 0.03	0.68 ± 0.03	−0.03 ± 0.02	0.0046	0.65
Ermellinata di Rovigo (24)	0.26	79	2.93 ± 0.34	2.79	1.87 ± 0.19	0.32 ± 0.06	0.37 ± 0.07	0.15 ± 0.03	0.0002	0.83
Livorno Bianca (44)	0.14	79	1.86 ± 0.14	1.80	1.37 ± 0.10	0.17 ± 0.04	0.22 ± 0.05	0.20 ± 0.05	0.0001	0.91
Livorno Nera (77)	0.20	100	2.57 ± 0.17	2.30	1.81 ± 0.12	0.43 ± 0.06	0.41 ± 0.05	−0.04 ± 0.03	0.1348	0.79
Mericanel della Brianza (99)	0.24	79	3.00 ± 0.50	2.59	1.86 ± 0.25	0.36 ± 0.07	0.36 ± 0.07	0.01 ± 0.03	0.0000	0.81
Millefiori di Lonigo (19)	0.36	100	3.29 ± 0.22	3.27	2.34 ± 0.16	0.53 ± 0.04	0.55 ± 0.03	0.05 ± 0.03	0.0028	0.74
Mugellese (22)	0.36	93	4.00 ± 0.47	3.83	2.43 ± 0.27	0.47 ± 0.05	0.52 ± 0.05	0.11 ± 0.03	0.0002	0.76
Padovana (21)	0.35	86	3.50 ± 0.48	3.45	2.59 ± 0.38	0.39 ± 0.07	0.49 ± 0.07	0.20 ± 0.03	0.0000	0.79
Pepoi (23)	0.18	79	2.64 ± 0.37	2.53	1.76 ± 0.19	0.30 ± 0.06	0.34 ± 0.07	0.09 ± 0.04	0.0031	0.84
Polverara (17)	0.38	93	4.14 ± 0.61	4.14	2.69 ± 0.35	0.39 ± 0.07	0.51 ± 0.08	0.21 ± 0.03	0.0000	0.79
Robusta Lionata (23)	0.25	86	2.86 ± 0.29	2.79	1.84 ± 0.16	0.34 ± 0.05	0.39 ± 0.06	0.12 ± 0.03	0.0003	0.83
Robusta Maculata (23)	0.18	79	2.14 ± 0.21	2.10	1.65 ± 0.17	0.26 ± 0.06	0.32 ± 0.06	0.16 ± 0.04	0.0034	0.86
Siciliana (42)	0.20	93	2.50 ± 0.17	2.19	1.68 ± 0.13	0.35 ± 0.06	0.36 ± 0.05	0.01 ± 0.05	0.0000	0.69
Valdarnese (30)	0.46	100	5.29 ± 0.60	4.80	3.05 ± 0.22	0.62 ± 0.05	0.65 ± 0.03	0.05 ± 0.03	0.0006	0.79
Romagnola (22)	0.31	100	3.29 ± 0.34	3.18	2.10 ± 0.25	0.42 ± 0.05	0.48 ± 0.03	0.12 ± 0.04	0.0074	0.83
Mean Breed	**0.31 ± 0.02**	**91**	**3.63 ± 0.13**	**3.31 ± 0.26**	**2.27 ± 0.09**	**0.43 ± 0.02**	**0.46 ± 0.02**	**0.09 ± 0.02**	**0.0000**	**0.78**

*N, sample size; PIC, polymorphic information content; P, percentage of polymorphic loci; Na, mean alleles number per locus; Rt, normalized allele size; the average number of alleles per locus corrected using the rarefaction method; Ne, effective alleles number (1/(sum pi^2^); Ho, observed heterozygosity (number of hets/N); He, expected heterozygosity (1 − sum pi^2^); *F*_*IS*_, heterozygote deficiency within breed (He − Ho)/He); He = 1 − (Ho/He); *P*, *P*-value for Global Hardy Weinberg test when H1 = heterozygote deficiency; *I*_*B*_, breed inbreeding. Mean self-coancestry, mean ± standard error.*

### Molecular Inbreeding

The Hind breed statistical analysis is shown in Table 2. Graphical output of Hind value distribution is depicted in Figure 1: whole dataset analysis, in red, showed a bell-shaped distribution, but the breed analysis, in black, showed a heterogeneous distribution of Hind, with a striking right-hand shift in Bionda Piemontese, Bianca di Saluzzo, and Valdarnese breeds and a worrying left-hand shift in Livorno Bianca. The *I*_B_ value confirmed a low (0.65) self-coancestry in Bionda Piemontese and Bianca di Saluzzo and a high self-coancestry (0.91) in *Livorno*. Mean *I*_B_ resulted to be 0.78 (Table 1).

**TABLE 2:** Breed mean Hind and statistical analysis.

	**AN**	**BP**	**BS**	**ER**	**LB**	**LN**	**MB**	**ML**	**MG**	**PD**	**PP**	**PV**	**RL**	**RM**	**SI**	**VA**	**RO**
M	0.45	0.69	0.69	0.32	0.17	0.43	0.36	0.53	0.47	0.39	0.30	0.39	0.34	0.26	0.35	0.62	0.42
Md	0.43	0.70	0.70	0.29	0.15	0.43	0.36	0.57	0.46	0.36	0.29	0.43	0.29	0.29	0.36	0.64	0.43
SD	0.14	0.13	0.14	0.09	0.09	0.12	0.11	0.13	0.14	0.13	0.13	0.12	0.13	0.08	0.11	0.13	0.13
SE	0.03	0.02	0.02	0.02	0.01	0.01	0.01	0.03	0.03	0.03	0.03	0.03	0.03	0.02	0.02	0.02	0.03
Min	0.21	0.43	0.31	0.14	0.00	0.14	0.14	0.29	0.14	0.14	0.00	0.14	0.14	0.07	0.07	0.36	0.14
Max	0.71	0.93	1.00	0.50	0.36	0.64	0.57	0.79	0.64	0.64	0.50	0.57	0.57	0.36	0.57	0.86	0.64

*AN, Ancona; BP, Bionda Piemontese; BS, Bianca di Saluzzo; ER, Ermellinata di Rovigo; LB, Livorno Bianca; LN, Livorno Nera; MB, Mericanel della Brianza; ML, Millefiori di Lonigo; MG, Mugellese; PD, Padovana; PP, Pepoi; PV, Polverara; RL, Robusta Lionata; RM, Robusta Maculata; SI, Siciliana; VA, Valdarnese; RO, Romagnola; M, mean; Md, median; SD, standard deviation; SE, standard error; Min, minimum value of Hind in breed; Max, maximum value of Hind in breed.*

### Molecular Kinship

Mean *K*_BW_ was 0.53, and individuals in the breed share on average 67% of their profile (PSA), indicating that they are immediate relatives. The *K*_G_ (0.27) and mean *K*_B__B_ (0.25) showed the differentiation and isolation between the breeds. *K*_BW_ ranged between 0.78 ± 0.01 (Livorno Bianca) and 0.31 ± 0.008 (Bionda Piemontese); *K*_BB_ ranged between 0.20 (Ermellinata), and 0.30 (Livorno Bianca and Romagnola).

*K*_BW_, m*K*_BB_, and PSA for each breed were plotted in Figure 2. In Livorno Bianca, it is possible to observe the higher value of *K*_BW_, PSA, and *K*_BB_, thus highlighting the close kinship between the individuals, and it looks like *K*_BB_ is not significantly different among breeds (the values vary between 0.20 and 0.30). On the contrary the Ermellinata shows the lower *K*_BB_. The Millefiori resulted to have the lowest value of PSA (0.33); this means that individuals share on average only 33% of genetic variability, even if this was not confirmed by the value *K*_BW_ corrected for sample size.

### Global Diversity and Breed Contributions

The fixation indices (*F*_IT_, *F*_ST_, and *F*_IS_) per locus are shown in Supplementary Table 1. Mean values bootstrapped adjusted for sampling and weighted for PIC information were considered to evaluate global biodiversity: with a mean value of 0.41 ± 0.005, the global heterozygosity deficit of individuals within the total population (*F*_IT_) was significantly high (*p* < 0.001). Fixation index of subpopulation in relation to the total population (*F*_ST_) per locus ranged from 0.023 at LEI192 to 0.043 at MCW0016 locus, with a mean of 0.34 ± 0.02 (*p* < 0.001). This indicates that about 35% of the total genetic variation in Italian chicken breed is explained by between-breed differences. The average inbreeding coefficient of individuals within the breeds, measured as *F*_IS_ value, was 0.09 ± 0.008 (*p* < 0.001). The contribution of each analyzed breed to global biodiversity was plotted in Figure 3; Bianca di Saluzzo (98.5%) and Bionda Piemontese (95.3%) had the greatest contribution to GD both in terms of genetic variability and allelic richness. They better offset the loss of overall variability due to GD_*B*_ loss with an increase of GB_*W*_ (+ 31.9% and + 28.6%, respectively). Except for Pepoi and Robusta Maculata that did not show any private alleles, all the other breeds contributed to global allelic richness, with 94 private alleles; this is a rather high number that confirms the results obtained by Soglia et al. (2017) in a survey carried out on two local chicken breeds. Bionda Piemontese adds 11% to global allele richness with 24 private alleles detected in 94% of individuals, whereas Bianca di Saluzzo adds 10% to global allele richness with 20 private alleles detected in 47% individuals; also, Siciliana carried out a 92.5% of contribution to GB but with a very low allele richness contribution with 1 private allele.

The analysis of breed impact on global genetic diversity is described in Table 3. The GD_*T*_ values underlined a little GD contribution of each Italian breed; for all the others, the values range from 2.38% (Bionda Piemontese) to 0.03% (Siciliana). Breeds that showed a negative impact on the GD_*W*_ were Bionda Piemontese, Bianca di Saluzzo, Valdarnese, Ancona, Millefiori, Mugellese, Polverara, Romagnola, and Padovana; therefore, they should be considered as important sources of genetic variability. On the other hand, Robusta Maculata, Livorno Bianca, Robusta Lionata, Ermellinata, Siciliana, Mericanel della Brianza, Pepoi, and Livorno Nera showed a negative impact on the global biodiversity related to breeds peculiarity.

**TABLE 3:** Breed impact on global diversity.

**Breed**	**GD**	**GD_W_**	**GD_B_**	**GD_T_**
Bionda Piemontese	0.68	–4.00	1.62	–2.38
Bianca di Saluzzo	0.69	–3.16	2.02	–1.14
Robusta Lionata	0.69	0.38	–1.23	–0.85
Valdarnese	0.69	–1.26	0.42	–0.84
Robusta Maculata	0.69	0.79	–1.43	–0.64
Ermellinata	0.69	0.50	–1.14	–0.64
Ancona	0.70	–0.48	0.22	–0.26
Polverana	0.70	–0.18	0.05	–0.12
Pepoi	0.70	0.65	–0.70	–0.05
Millefiori Lonigo	0.70	–0.36	0.31	–0.04
Siciliana	0.70	1.09	–1.07	0.03
Padovana	0.70	–0.13	0.16	0.03
Mugellese	0.70	–0.29	0.35	0.06
Romagnola	0.70	–0.05	0.34	0.29
Livorno Nera	0.70	1.11	–0.48	0.63
Livorno Bianca	0.71	2.67	–1.47	1.20
Mericanel della Brianza	0.71	2.73	–0.89	1.85

*GD, genetic diversity of the dataset after removing the breed; GD_*W*_, percentage of lost within-breed diversity; GD_*B*_, percentage of lost between-breeds diversity. GD_*T*_ = GD_*W*_ + GD_*B*_, the global breed extinction impact on genetic diversity, a positive GD_*T*_ value means that the remaining dataset increases the overall diversity. In red is the positive contribution to GD.*

### Conservation Risk Status

The analysis of risk extinction for Italian breeds pointed out that 65% of Italian breed showed an ERI value of 2.5 (Figure 4). Livorno Bianca turned out to be the breed with the lowest genetic variability and the highest ERI (3.53) among the Italian chicken breeds. For this breed, but also for others (Mugellese, Padovana, Pepoi, Polverara, Robusta Lionata, Robusta Maculata, and Valdarnese), it was possible to observe a potential heterozygous, where the He is higher than the Ho, which could be recovered with a careful mating management. On the other hand, Robusta Maculata and Ermellinata resulted to have a high ERI value (3.17 and 2.96, respectively), with few possibilities to increase their individual variability. The highest variability values were found in Bianca di Saluzzo and Bionda Piemontese, although the percentage of alleles with a frequency lower than 5% was high, so the genetic drift could reduce the variability of these two breeds very quickly.

## Discussion

### Prior Research

The autochthonous chicken breeds biodiversity preservation is a topic of rising importance in recent time; indeed, some investigations were already devoted to this aim not only in Italy (Ceccobelli et al., 2015 and Sartore et al., 2016), but all over the world (Tadano et al., 2007; Bodzsar et al., 2009; Zanetti et al., 2011; Malomane et al., 2019). The objectives of all these studies were to assess the risk status of the breeds and therefore to provide a starting point for suggesting proper mating schemes; the final goal is to prevent the increasing of the inbreeding. All these surveys were carried out with the aid of microsatellite loci, namely, with the set of markers recommended by the ISAG that are still a reliable tool for this kind of investigations. The set of 14 microsatellite markers selected in Soglia et al. (2017) resulted polymorphic in Italian chicken breeds and showed a PIC equal to 0.66, with a total number of alleles equal to 209 and a mean value for locus of 14.93 ± 2.62 and 3.62 ± 0.13 within breed. Consequently, the informative content of these 14 microsatellites is similar to that reported by Zanetti et al. (2011) in a survey carried out on other Italian poultry breeds. In this context, our investigation provides a current picture of the genetic diversity and thus of the potential risk status of 17 local chicken breeds reared in North, Central, and South of Italy. Our investigation provides further information about the contribution of the Italian autochthonous chicken breeds to Italian poultry biodiversity and moreover formulates the ERI to quantify the breed extinction risk based on molecular data. This formula combines different genetic parameters linked to homozygosity excess, kinship, and within-breed diversity; furthermore, the ERI takes into account individual self-coancestry. The evaluation of both genetic variability and risk status is the first step for mating plans managing with the aim of reducing the effect of genetic drift in local breeds, some of them currently in a critical status due to their small size.

### Potential Shortcomings

Different parameters were investigated with the aim of recovering a real and reliable picture of genetic variation, and risk status of 17 autochthonous chicken breeds. All the animals investigated in this survey are reared in conservation centers aimed to the preservation of these local genetic resources (Figure 5).

Generally, the within-breed genetic diversity of the studied chicken breeds is low (Ho = 0.44 and *K*_BW_ = 0.53) similar to the results obtained by Zanetti et al. (2011) in a survey carried out on a group of native chicken breed from Veneto region and by Ceccobelli et al. (2013) in a study carried out on five native chicken breeds reared in Middle Italy. He and Ho were markedly different among the breeds as reported by Cendron et al. (2021), wavering between 0.17 and 0.69 in Bianca di Saluzzo and Livorno Bianca, respectively. The average Ho in some breeds was lower than that reported in previous studies based on microsatellite markers (Zanetti et al., 2010, 2011) and more similar with SNP genetic variability reported by Cendron et al. (2020, 2021); this Ho deficit could be due to an increase in inbreeding linked to a reduction in the number of individuals within the breed over the years.

This is highlighted by a small number of alleles per locus detected in many breeds (6.71–1.86; mean = 3.62) but also by low heterozygosity estimates (0.69–0.17), and high within-breed inbreeding (0.67–0.31). The Ne was low in all breeds (<3.57) due to the presence of low-frequency alleles, 20% of which with frequency < 5% that can be easily lost by genetic drift, thus reducing the genetic variability of the breeds, and increasing their risk of extinction. Given the fact that the mean Ho and *F*_is_ indicate an excess of homozygosity in several breeds, consistently the levels of self-coancestry were also high. Global heterozygosity deficit of individuals within the total population (*F*_IT_ = 0.40) was significantly high (*p* < 0.001). The fixation index of breed in relation to the total population (*F*_ST_) with mean 0.35 ± 0.004 (*p* < 0.001) indicates that about 35% of the total genetic variation in autochthonous Italian chicken breeds is explained by between-breed differences, in comparison to 22% observed by Ceccobelli et al. (2015) in a group of 16 local chicken breeds originating from different countries, namely, Italy, Spain, Serbia, Albania, and Malta. Similar values are reported by Strillacci et al. (2017) in some Italian autochthonous breeds, whereas Zanetti et al. (2011) detected an *F*_ST_ = 0.41 in autochthonous breeds from Veneto region. The Livorno Bianca and Romagnola showed higher *K*_BB_; on the contrary, the Ermellinata showed the lower *K*_BB_. In molecular inbreeding, the breed analysis distribution highlighted a heterogeneous distribution of Hind, with a remarkable right-hand shift in Bionda Piemontese, Bianca di Saluzzo, and Valdarnese. Bianca di Saluzzo (98.5) and Bionda Piemontese (95.3) provided the greatest contribution to global biodiversity and showed the highest variability values, although the percentage of alleles with a frequency lower than 5% was high, so the genetic drift could reduce the variability of these two breeds very quickly. A non-significant *F* negative value observed in Bionda Piemontese and Bianca di Saluzzo could be related to the fact that in the last years these breeds were submitted to targeted mating schemes with the aim of preserving individual variability, and thus, the inbreeding effect is negligible (Soglia et al., 2018). The molecular variability of our local breeds can be regarded as an important reservoir of genetic variation (Muir et al., 2008); the Valdarnese as well showed high variability values: great levels for Na and Ho and good Hind distribution; the low proportion of shared allele among individuals (PSA = 0.48) showed a high within-breed variability confirmed by a low kinship. Also, its contribution to allele richness (0.4%), as private alleles, was among the highest, and found in 40% of individuals. The SNP analysis carried out by Cendron et al. (2020) revealed a higher Ho value in the Valdarnese compared to the other breeds investigated, and Ceccobelli et al. (2015) pointed out its high within-breed variability by mitochondrial haplotype investigation. Because of its own peculiarity, the extinction of the Valdarnese breed should have a negative effect on the global variability similar to the extinction of Ermellinata and just lower than the extinction of Bionda Piemontese, Bianca di Saluzzo, and Robusta Lionata. The lowest contribution to overall genetic diversity was provided by Livorno Bianca. The molecular kinship results provided important information about the situation of this breed: the high value of *I*_B_, *K*_BW_, PSA, and a worrying left-hand shift of Hind distribution should be taken carefully into account. The risk status analysis revealed that Livorno Bianca turned out to be the breed with the lowest genetic variability and the highest ERI, even if it was possible to observe an heterozygosity, where the He is higher than the Ho, which could be recovered with a careful mating plain management; this finding is in accordance with Strillacci et al. (2017), who reported a relevant value of inbreeding in Livorno related to the small size of the population under conservation for many years. Cartoni-Mancinelli et al. (2020) and Castillo et al. (2021) confirmed the small number of breeders of Livorno and the subdivision in small populations, with a likely increase of homozygosity.

In addition, despite the fact of presenting a high number of alleles, also in Robusta, Pepoi, and Polverara, the genetic management requires a particular attention due to the high inbreeding coefficient and within-breed kinship. Likewise, Siciliana shows a very low variability and a high kinship; therefore, mating schemes with other Siciliana genetic lines are required. In Cendron et al. (2020) with runs of homozygosity analysis, pointed out in eight autochthonous breeds from Veneto region a low heterozygosity index, and a relevant inbreeding coefficient. As reported by Castillo et al. (2021), the risk status “endangered-maintained” or “vulnerable” has been attributed to these breeds by FAO.

The Robusta Maculata and Ermellinata as well resulted to have high ERI (3.17 and 2.96, respectively) and low variability value with few possibilities to increase their individual variability. Given this fact, their conservation status could be worrying as already seen in other local breeds (De Marchi et al., 2006; Zanetti et al., 2011; Ceccobelli et al., 2013; Strillacci et al., 2017; Cendron et al., 2020).

The Millefiori had the lowest value of PSA (0.33); this means that individuals share on average only the 33% of genetic variability, even if this result was not confirmed by the kinship value corrected for sample size. This could be justified by an effect resulting from the different weight given to the sharing of alleles in the calculation of the MolKin respect to the PSA and the reduced number of sampled subjects. That could be better understood through further studies about breed structure and genetic management of mating.

In genetic diversity contribution analysis, breeds that showed a negative impact on the GD_W_ were Bionda Piemontese, Bianca di Saluzzo, Valdarnese, Ancona, Millefiori, Mugellese, Polverara, and Padovana; therefore, they should be considered as important sources of genetic variability. On the other hand, Robusta Maculata, Livorno Bianca, Robusta Lionata, Ermellinata, Siciliana, Mericanel della Brianza, Pepoi, and Livorno Nera showed a negative impact on the global biodiversity related to breeds peculiarity. This finding confirms the results obtained by Strillacci et al. (2017) in a survey carried out on six Italian chicken native breeds; even in that case, Livorno and Siciliana breeds showed a low genetic variation due to their small size, and Mericanel della Brianza breed had both He and Ho lower than that obtained by Tadano et al. (2008) on Japanese Bantam breeds. According to Medugorac et al. (2011) and Ginja et al. (2013, 2018), the conservation objectives may vary, depending on the final aim of these local breeds preservation: in a short-term strategy, a high heterozygosis level should be preserved (GD_*W*_), whereas in a long-term strategy, the allelic richness and breed differentiation are crucial. Summarizing the obtained results, Italian autochthonous breeds provide an important contribution to poultry genetic variability due to their unique genetic pattern, compared to autochthonous breeds of other countries (Bodzsar et al., 2009; Abebe et al., 2015). Moreover, Italian breeds show low genetic diversity and high self-inbreeding. Furthermore, it is possible to observe a potential heterozygosity, where He is higher than Ho, which could be recovered with a careful mating management. The introduction of different genetic lines and the use of mating schemes are strongly recommended as part of a conservation strategy aimed to limit inbreeding increase.

## Data Availability Statement

The datasets presented in this study can be found in online repositories. The names of the repository/repositories and accession number(s) can be found below: https://figshare.com/ and https://doi.org/10.6084/m9.figshare.14685882.

## Ethics Statement

The animal study was reviewed and approved by Bioethical Committee of the University of Turin (prot. no. 451944).

## Author Contributions

DS, SS, and AS: conceptualization, methodology, and original draft preparation. MM, NI, AB, CC, and FC: samples collection. SM, CB, SD, and MP: sample analysis. DS and SS: data analyses. EL, FP, MC, and AC: writing – review, and editing. AS, PS, and MC: supervision. AS and SC: project administration and funding acquisition. All authors have read and agreed to the published version of the manuscript.

## Conflict of Interest

The authors declare that the research was conducted in the absence of any commercial or financial relationships that could be construed as a potential conflict of interest.

## Publisher’s Note

All claims expressed in this article are solely those of the authors and do not necessarily represent those of their affiliated organizations, or those of the publisher, the editors and the reviewers. Any product that may be evaluated in this article, or claim that may be made by its manufacturer, is not guaranteed or endorsed by the publisher.

## Footnotes

1
https://www.pollitaliani.it/en/project/

2
https://aviandiv.fli.de/
