# Supplementary material for: Genetic Diversity of 17 Autochthonous Italian Chicken Breeds and Their Extinction Risk Status
Source: Front Genet. 2021 Sep 14;12:715656. doi: 10.3389/fgene.2021.715656 (PMC8477013; doi:10.3389/fgene.2021.715656)
Supplement: Supplementary file 1 [file Data_Sheet_1.PDF]

Global Hardy-Weinberg tests [Score (U) test]

File /web/genepop/tmp/193249/193249

Number of populations : 17

Number of loci :   14

Markov chain parameters for all tests :

Dememorization :   10000

Batches :   500

Iterations per batch : 5000

Hardy Weinberg test when H1= heterozygote deficit

****

==

Results by population (test multi-locus)

==

Population	P-val	S.E.	switches (ave.)
AN	0.0000	0.0000	600762.36
BP	0.0009	0.0001	617172.07
BS	0.0046	0.0003	453508.00
ER	0.0002	0.0000	630101.09
LVB	0.0001	0.0000	1209354.30
LVN	0.1348	0.0010	1393703.85
MB	0.0000	0.0000	1153244.10
ML	0.0028	0.0001	825681.36
MUG	0.0002	0.0000	610577.31

PD	0.0000	0.0000	678936.50
PP	0.0031	0.0001	721059.36
PV	0.0000	0.0000	546578.42
RL	0.0003	0.0000	723361.25
RM	0.0034	0.0001	1034305.40
SIC	0.0000	0.0000	914340.67
VAL	0.0006	0.0001	561383.00
ROM	0.0074	0.0002	744742.36

==

Results by locus (test multi-population)

==

Locus	P-val	S.E.	switches (ave.)
-----------	------	------	--------
LEI0166	0.2243	0.0014	776256.20
LEI0192	0.0000	0.0000	551239.06
LEI0228	0.0000	0.0000	666713.62
LEI0258	0.0001	0.0000	518414.24
MCW0034	0.0430	0.0010	673560.64
MCW0069	0.8570	0.0013	828265.42
MCW0078	0.1246	0.0012	907861.67
MCW0104	0.0003	0.0001	552023.20
ADL0278	0.0000	0.0000	821863.38
MCW0016	0.5564	0.0037	844614.60
MCW0020	0.9725	0.0006	962023.57
MCW0037	0.0000	0.0000	913276.25
MCW0206	0.7870	0.0027	913407.69
MCW0222	0.0191	0.0004	981985.15

==

Result for all locus and all populations

==

P-val   S.E.   switches (ave.)

------ ------ --------

0.0000 0.0000 773769.28

Normal ending.
