# Supplementary material for: Genetic Diversity of 17 Autochthonous Italian Chicken Breeds and Their Extinction Risk Status
Source: Front Genet. 2021 Sep 14;12:715656. doi: 10.3389/fgene.2021.715656 (PMC8477013; doi:10.3389/fgene.2021.715656)
Supplement: Supplementary file 2 [file Data_Sheet_2.PDF]

SUBPOP	GD	Internal_Diversity	Mean_Distance	Loss/Gain	Pe_Int_Diversity	Pe_Divergence	Petit(34) (%)
Ancona	0,70	-0,48	0,22	-0,26	1,01	-0,04	0,97
BiondaPiemontese	0,68	-4,00	1,62	-2,38	1,76	1,10	2,86
BiancaSaluzzo	0,69	-3,16	2,02	-1,14	2,39	1,53	3,92
Ermellinata	0,69	0,50	-1,14	-0,64	-0,58	2,38	1,80
LivornoB	0,71	2,67	-1,47	1,20	-1,69	1,80	0,10
LivornoN	0,70	1,11	-0,48	0,63	-1,13	-1,12	-2,25
Mericanel della Brianza	0,71	2,73	-0,89	1,85	-0,81	-1,01	-1,82
Millefiori Lonigo	0,70	-0,36	0,31	-0,04	-0,05	-0,08	-0,13
Mugellese	0,70	-0,29	0,35	0,06	0,58	0,16	0,74
Padovana	0,70	-0,13	0,16	0,03	0,15	1,00	1,15
Pepoi	0,70	0,65	-0,70	-0,05	-0,87	0,97	0,10
Polverana	0,70	-0,18	0,05	-0,12	0,92	-0,22	0,71
Robusta Lionata	0,69	0,38	-1,23	-0,85	-0,58	2,13	1,55
Robusta Maculata	0,69	0,79	-1,43	-0,64	-1,36	1,95	0,59
Siciliana	0,70	1,09	-1,07	0,03	-1,26	0,91	-0,34
Valdarnese	0,69	-1,26	0,42	-0,84	1,66	0,02	1,68
Romagnola	0,70	-0,05	0,34	0,29	-0,14	-0,08	-0,22
