# Supplementary material for: Genetic Diversity of 17 Autochthonous Italian Chicken Breeds and Their Extinction Risk Status
Source: Front Genet. 2021 Sep 14;12:715656. doi: 10.3389/fgene.2021.715656 (PMC8477013; doi:10.3389/fgene.2021.715656)
Supplement: Supplementary file 3 [file Data_Sheet_3.PDF]

FreeNA,

Input file = 17razze.txt

Output file = frena.r

Number of populations = 17

Number of loci = 14

Estimating null allele frequency using the EM algorithm (Dempster et al. 1977)

Locus	Pop	Estimate of null allele frequency
1	1	0.02117
1	2	0.00000
1	3	0.00001
1	4	0.00100
1	5	0.00001
1	6	0.00015
1	7	0.00001
1	8	0.00000
1	9	0.00001
1	10	0.10933
1	11	0.04589
1	12	0.08357
1	13	0.10984
1	14	0.26087
1	15	0.00001
1	16	0.00002
1	17	0.00000

2	1	0.22464
2	2	0.00000
2	3	0.05634
2	4	0.03067
2	5	0.13642
2	6	0.03329
2	7	0.00100
2	8	0.01764
2	9	0.00000
2	10	0.00000
2	11	0.16039
2	12	0.10747
2	13	0.02005
2	14	0.15209
2	15	0.09128
2	16	0.00000
2	17	0.06175
3	1	0.16403
3	2	0.29042
3	3	0.13806
3	4	0.00100
3	5	0.00002
3	6	0.16495
3	7	0.14697
3	8	0.26898
3	9	0.12069
3	10	0.08650
3	11	0.00001

3	12	0.00979
3	13	0.07348
3	14	0.10231
3	15	0.00001
3	16	0.25558
3	17	0.25586
4	1	0.14972
4	2	0.00000
4	3	0.00000
4	4	0.04398
4	5	0.30208
4	6	0.00000
4	7	0.06651
4	8	0.09273
4	9	0.05619
4	10	0.12023
4	11	0.00000
4	12	0.08649
4	13	0.05215
4	14	0.00000
4	15	0.10533
4	16	0.03960
4	17	0.00000
5	1	0.10198
5	2	0.00001
5	3	0.00000
5	4	0.03635
5	5	0.00008

5	6	0.00005
5	7	0.00100
5	8	0.00002
5	9	0.12224
5	10	0.00000
5	11	0.05864
5	12	0.03808
5	13	0.00100
5	14	0.00004
5	15	0.12921
5	16	0.00000
5	17	0.00003
6	1	0.00000
6	2	0.00000
6	3	0.00000
6	4	0.00100
6	5	0.00100
6	6	0.01089
6	7	0.00100
6	8	0.00000
6	9	0.00001
6	10	0.09336
6	11	0.00100
6	12	0.14657
6	13	0.00000
6	14	0.03613
6	15	0.00100
6	16	0.00000

6	17	0.07598
7	1	0.01120
7	2	0.01561
7	3	0.00046
7	4	0.00001
7	5	0.00100
7	6	0.00001
7	7	0.01528
7	8	0.02835
7	9	0.00000
7	10	0.00000
7	11	0.12281
7	12	0.07639
7	13	0.00100
7	14	0.00000
7	15	0.00001
7	16	0.05328
7	17	0.00001
8	1	0.02137
8	2	0.00000
8	3	0.00000
8	4	0.15770
8	5	0.00001
8	6	0.00001
8	7	0.08332
8	8	0.00001
8	9	0.15145
8	10	0.19346

8	11	0.00004
8	12	0.00097
8	13	0.24942
8	14	0.00100
8	15	0.00008
8	16	0.05472
8	17	0.05604
9	1	0.09646
9	2	0.06288
9	3	0.01952
9	4	0.10863
9	5	0.00008
9	6	0.01611
9	7	0.04253
9	8	0.07164
9	9	0.01186
9	10	0.17608
9	11	0.11367
9	12	0.16609
9	13	0.00053
9	14	0.00829
9	15	0.00000
9	16	0.05042
9	17	0.09091
10	1	0.00000
10	2	0.01590
10	3	0.00000
10	4	0.00000

10	5	0.00001
10	6	0.00508
10	7	0.00000
10	8	0.02842
10	9	0.07834
10	10	0.00100
10	11	0.00000
10	12	0.00100
10	13	0.04748
10	14	0.00001
10	15	0.00000
10	16	0.00001
10	17	0.00000
11	1	0.00000
11	2	0.00000
11	3	0.00008
11	4	0.00001
11	5	0.00100
11	6	0.00000
11	7	0.00000
11	8	0.00000
11	9	0.07155
11	10	0.19233
11	11	0.00100
11	12	0.08019
11	13	0.00181
11	14	0.00100
11	15	0.00001

11	16	0.00001
11	17	0.15790
12	1	0.19804
12	2	0.15101
12	3	0.01773
12	4	0.13288
12	5	0.04057
12	6	0.00002
12	7	0.08053
12	8	0.00000
12	9	0.00002
12	10	0.01930
12	11	0.00058
12	12	0.20029
12	13	0.00001
12	14	0.00100
12	15	0.16861
12	16	0.11777
12	17	0.01797
13	1	0.00001
13	2	0.00000
13	3	0.00000
13	4	0.05519
13	5	0.08739
13	6	0.00000
13	7	0.00000
13	8	0.00000
13	9	0.02760

13	10	0.00001
13	11	0.00002
13	12	0.00003
13	13	0.00000
13	14	0.12864
13	15	0.05500
13	16	0.00000
13	17	0.00000
14	1	0.11222
14	2	0.00005
14	3	0.02612
14	4	0.02437
14	5	0.00738
14	6	0.00000
14	7	0.00019
14	8	0.00000
14	9	0.00100
14	10	0.00100
14	11	0.00100
14	12	0.14386
14	13	0.03045
14	14	0.00004
14	15	0.00001
14	16	0.01852
14	17	0.10538
