# Supplementary material for: Genetic Diversity of 17 Autochthonous Italian Chicken Breeds and Their Extinction Risk Status
Source: Front Genet. 2021 Sep 14;12:715656. doi: 10.3389/fgene.2021.715656 (PMC8477013; doi:10.3389/fgene.2021.715656)
Supplement: Supplementary file 4 [file Data_Sheet_4.PDF]

Locus	PIC	Na	Ne	Ho	He	F_{IS}	F_{IT}	F_{ST}	*P*
**LEI0166**	0.57	5	2.70	0.43	0.63	0.32	0.38	0.33	0.2243
**LEI0192**	0.71	28	3.71	0.47	0.73	0.35	0.34	0.23	0.0000
**LEI0228**	0.87	24	8.26	0.30	0.88	0.66	0.63	0.42	0.0000
**LEI0258**	0.92	39	12.78	0.58	0.92	0.37	0.40	0.33	0.0001
**MCW0034**	0.76	16	4.62	0.48	0.78	0.39	0.41	0.37	0.0430
**MCW0069**	0.45	10	1.89	0.36	0.47	0.24	0.28	0.30	0.8570
**MCW0078**	0.53	7	2.57	0.45	0.61	0.26	0.30	0.31	0.1246
**MCW0104**	0.73	21	4.08	0.43	0.76	0.42	0.47	0.37	0.0003
**ADL0278**	0.78	12	5.25	0.52	0.81	0.36	0.39	0.30	0.0000
**MCW0016**	0.58	12	2.76	0.45	0.64	0.29	0.38	0.43	0.5564
**MCW0020**	0.67	8	3.59	0.50	0.72	0.31	0.42	0.40	0.9725
**MCW0037**	0.55	8	2.61	0.33	0.62	0.46	0.45	0.31	0.0000
**MCW0206**	0.72	13	4.23	0.51	0.76	0.34	0.39	0.41	0.7870
**MCW0222**	0.42	6	1.85	0.36	0.46	0.22	0.32	0.29	0.0191
**Mean**	0.66	15	4.35	0.44	0.70	0.36	0.40	0.34	0.0000
SE	*0.04*	*2.62*	*0.78*	*0.02*	*0.04*	*0.03*	*0.02*	*0.02*	*0.0000*

Table 1 Locus microsatellite parameters: PIC= polymorphic information content, Na= No. of alleles, Ne =No. of effective alleles 1 / (Sum pi2), Ho=Observed Heterozygosity (No. of Hets / N sample size), He=Expected Heterozygosity (1 - Sum pi2), FIS= heterozygote deficiency within breed ((He - Ho) / He); He = 1 - (Ho / He), HWE= Test for Hardy-Weinberg Equilibrium (ns=non-significant, * P<0.05, ** P<0.01, *** P<0.001), FIT = heterozygote deficiency in the total breed ((Ht - Mean Ho) / Ht); FST= heterozygote deficiency due to breed subdivision (Ht - Mean He) / Ht); Ht = total expected Heterozygosity = 1 - Sum tpi2 where tpi is the frequency of the ith allele for the total and Sum tpi2 is the sum of the squared total allele frequencies. P= P-value for Global Hardy Weinberg test when H1= heterozygote deficit

BREED	AN	BP	BS	ER	LB	LN	MB	ML	MG	PD	PP	PV	RL	RM	SI	VA	RO	K_{BB}
**Ancona (AN)**	0.43																	0.25
**Bionda Piemontese (BP)**	0.22	0.31																0.21
**Bianca di Saluzzo (BS)**	0.27	0.23	0.32															0.24
**Ermellinata (ER)**	0.21	0.20	0.20	0.61														0.20
**Livorno bianca (LB)**	0.28	0.22	0.29	0.25	0.78													0.30
**Livorno nera (LN)**	0.18	0.23	0.21	0.17	0.41	0.59												0.26
**Mericanel della Brianza (MB)**	0.25	0.20	0.24	0.29	0.34	0.32	0.62											0.27
**Millefiori Lonigo (ML)**	0.26	0.25	0.25	0.18	0.32	0.24	0.27	0.46										0.27
**Mugellese (MG)**	0.28	0.23	0.25	0.22	0.35	0.26	0.30	0.32	0.45									0.27
**Padovana (PD)**	0.26	0.22	0.25	0.16	0.28	0.28	0.28	0.26	0.26	0.48								0.26
**Pepoi (PP)**	0.30	0.19	0.25	0.24	0.26	0.26	0.33	0.26	0.24	0.28	0.64							0.24
**Polverara (PV)**	0.27	0.23	0.25	0.19	0.26	0.28	0.26	0.27	0.26	0.37	0.24	0.48						0.26
**Robusta Lionata (RL)**	0.24	0.16	0.16	0.16	0.27	0.23	0.19	0.22	0.24	0.21	0.13	0.23	0.61					0.21
**Robusta Maculata (RM)**	0.20	0.16	0.16	0.18	0.26	0.20	0.25	0.31	0.30	0.18	0.15	0.15	0.37	0.67				0.22
**Siciliana (SI)**	0.23	0.21	0.25	0.21	0.30	0.31	0.28	0.30	0.28	0.35	0.29	0.34	0.13	0.14	0.66			0.26
**Valdarnese (VA)**	0.24	0.23	0.21	0.18	0.25	0.23	0.22	0.27	0.23	0.22	0.23	0.21	0.14	0.20	0.18	0.35		0.22
**Romagnola (RO)**	0.32	0.23	0.29	0.23	0.42	0.28	0.33	0.35	0.30	0.33	0.26	0.31	0.24	0.24	0.32	0.27	0.52	0.30

Table 2 Molecular Kinship analysis: kinship between breeds is reported as matrix table, in the last column the mean between breeds kinship (K_{BB}) and on diagonals within breed kinship (K_{BW}).

	AN	*BP*	*BS*	*ER*	*LB*	*LN*	*MB*	*ML*	*MG*	*PD*	*PP*	*PV*	*RL*	*RM*	*SI*	*VA*	*RO*	*N*
LEI0166	0.02	0.00	0.00	0.00	0.00	0.00	0.00	0.00	0.00	0.11	0.05	0.08	0.11	0.26	0.00	0.00	0.00	**4**
LEI0192	0.22	0.00	0.06	0.03	0.14	0.03	0.00	0.02	0.00	0.00	0.16	0.11	0.02	0.15	0.09	0.00	0.06	**5**
LEI0228	0.16	0.29	0.14	0.00	0.00	0.16	0.15	0.27	0.12	0.09	0.00	0.01	0.07	0.10	0.00	0.26	0.26	**10**
LEI0258	0.15	0.00	0.00	0.04	0.30	0.00	0.07	0.09	0.06	0.12	0.00	0.09	0.05	0.00	0.11	0.04	0.00	**4**
MCW0034	0.10	0.00	0.00	0.04	0.00	0.00	0.00	0.00	0.12	0.00	0.06	0.04	0.00	0.00	0.13	0.00	0.00	**3**
MCW0069	0.00	0.00	0.00	0.00	0.00	0.01	0.00	0.00	0.00	0.09	0.00	0.15	0.00	0.04	0.00	0.00	0.08	**1**
MCW0078	0.01	0.02	0.00	0.00	0.00	0.00	0.02	0.03	0.00	0.00	0.12	0.08	0.00	0.00	0.00	0.05	0.00	**2**
MCW0104	0.02	0.00	0.00	0.16	0.00	0.00	0.08	0.00	0.15	0.19	0.00	0.00	0.25	0.00	0.00	0.05	0.06	**4**
ADL0278	0.10	0.06	0.02	0.11	0.00	0.02	0.04	0.07	0.01	0.18	0.11	0.17	0.00	0.01	0.00	0.05	0.09	**4**
MCW0016	0.00	0.02	0.00	0.00	0.00	0.01	0.00	0.03	0.08	0.00	0.00	0.00	0.05	0.00	0.00	0.00	0.00	**0**
MCW0020	0.00	0.00	0.00	0.00	0.00	0.00	0.00	0.00	0.07	0.19	0.00	0.08	0.00	0.00	0.00	0.00	0.16	**1**
MCW0037	0.20	0.15	0.02	0.13	0.04	0.00	0.08	0.00	0.00	0.02	0.00	0.20	0.00	0.00	0.17	0.12	0.02	**5**
MCW0206	0.00	0.00	0.00	0.06	0.09	0.00	0.00	0.00	0.03	0.00	0.00	0.00	0.00	0.13	0.06	0.00	0.00	**1**
MCW0222	0.11	0.00	0.03	0.02	0.01	0.00	0.00	0.00	0.00	0.00	0.00	0.14	0.03	0.00	0.00	0.02	0.11	**3**
N	**6**	**6**	**1**	**3**	**2**	**1**	**1**	**1**	**3**	**5**	**3**	**4**	**2**	**4**	**3**	**2**	**3**	

Table 3 Estimated null allele frequency using the EM algorithm (Dempster et al. 1977) with FreeNA N=number of loci/breed with a frequency of null allele >0.10
